# Optimization of Supplemental LED Spectral Quality and Light Dose for Enhancing Biomass and Essential Oil Yield of *Ocimum gratissimum* L. Under Net House Condition

**DOI:** 10.3390/molecules30183753

**Published:** 2025-09-15

**Authors:** Ha Thi Thu Chu, Thi Nghiem Vu, Thuy Thi Thu Dinh, Phat Tien Do, Tran Quoc Tien, Quang Cong Tong, Quyen Thi Ha, Khanh Quoc Tran, William N. Setzer

**Affiliations:** 1 Institute of Biology, Vietnam Academy of Science and Technology, 18 Hoang Quoc Viet, Nghia Do, Hanoi 10072, Vietnam; dtphat@ib.ac.vn; 2 Institute of Materials Science, Vietnam Academy of Science and Technology, 18 Hoang Quoc Viet, Nghia Do, Hanoi 10072, Vietnam; vtnghiem@ims.vast.ac.vn (T.N.V.); tientq@ims.vast.ac.vn (T.Q.T.); congtq@ims.vast.ac.vn (Q.C.T.); 3 Institute of Chemistry, Vietnam Academy of Science and Technology, 18 Hoang Quoc Viet, Nghia Do, Hanoi 10072, Vietnam; dttthuy@ich.vast.vn; 4 Faculty of Agricultural Technology, VNU University of Engineering and Technology, Vietnam National University Hanoi, 144 Xuan Thuy, Cau Giay, Hanoi 10053, Vietnam; quyenht@vnu.edu.vn; 5 Laboratory of Adaptive Lighting Systems and Visual Processing, Technical University of Darmstadt, Hochschulstr. 4a, 64289 Darmstadt, Germany; 6 Department of Chemistry, University of Alabama in Huntsville, Huntsville, AL 35899, USA; setzerw@uah.edu; 7 Aromatic Plant Research Center, 230 N 1200 E, Suite 100, Lehi, UT 84043, USA

**Keywords:** clove basil, *Essential oil*, eugenol, germacrene D, light spectra, *Ocimum gratissimum* L., optimal supplemental lighting treatment, phenylpropanoids, (Z)-β-ocimene

## Abstract

This study investigated optimal supplemental multispectral light-emitting diode (LED) conditions for the growth and essential oil accumulation of *Ocimum gratissimum* L. (OG) cultivated in a net house over 15 weeks. We hypothesized that supplemental lighting could increase biomass while affecting oil yield or vice versa. Nine lighting treatments were established, combining red (R) and blue (B) with ultraviolet-A (UV-A), green (G), or far-red (Fr) lights, applied for 4, 6, or 8 h/night at 80–120 µmol·m^−2^·s^−1^. Essential oils were analyzed by GC/MS-FID, revealing 21–28 compounds, dominated by phenylpropanoids (59.4–71.2%). Eugenol (58.5–69.8%), (*Z*)-β-ocimene (10.2–12.1%), and germacrene D (7.6–12.1%) were the major constituents. The oils showed weak antimicrobial activity against *Candida albicans*. All lighting treatments significantly enhanced fresh biomass and oil yield (*p* < 0.001) compared with the control. The optimal treatment (F2; R, B, and UV-A lights at a photon flux ratio of 71:20:9; 100 µmol·m^−2^·s^−1^, 6 h/night) yielded the highest fresh biomass (13.07 ± 0.71 Mg/ha), essential oil (31.39 ± 1.71 L/ha), and eugenol (21.09 ± 1.15 L/ha). These findings demonstrate the strong influence of spectral composition and exposure duration on OG productivity and highlight the potential of tailored LED strategies to improve both biomass and oil quality in cultivation systems.

## 1. Introduction

Light is a key environmental cue regulating plant growth and development, acting through a shared signaling pathway [1]. Among the various wavelengths, red (R) and blue (B) lights are the most efficient for driving photosynthesis of plants, because chlorophyll pigments primarily absorb light in these two wavelength regions. Other spectral regions such as ultraviolet-A (UV-A), green (G), and far-red (Fr) lights also act as important environmental signals for plants [2]. Supplementation of UV-A light has been found to stimulate photosynthesis and enhance the biosynthesis of bioactive secondary metabolites such as phenolics, flavonoids, etc. [3]. For instance, radiations in the wavelength range between UV-A and visible violet (385 nm, 30 W·m^−2^) applied for five days increased both yield and phenolic content in kale (*Brassica oleracea* var. *acephala*) [4]. Similarly, lettuce (*Lactuca sativa*) exposed to UV-A (10, 20, and 30 μmol·m^−2^·s^−1^, 365 nm) light for 13 days exhibited greater fresh and dry weight, leaf area, and antioxidant levels than those without UV-A [5]. In tomatoes (*Solanum lycopersicum*), the combination of R (215 µmol·m^−2^·s^−1^) and UV-A (35 µmol·m^−2^·s^−1^) light significantly boosted flavonoid content compared with R light alone [6]. The composition of photosynthetic pigments in coleus (*Coleus blumei*) was found to be higher under an R (660 nm):B regime than under an R(635 nm):B lighting condition [7].

Although G light is often underestimated due to its lower photosynthetic efficiency, it can penetrate deep into leaf tissues, contributing to increased carbon accumulation and crop yield [8]. In addition to being absorbed, G light also promotes and regulates physiological responses, improves stomatal function, refines canopy structure, and optimizes resource use efficiency [9]. Notably, Kim et al. [10] reported that adding 24% G light to R:B light-emitting diode (LED) increased lettuce (*Lactuca sativa*) biomass by 47%, despite identical total photosynthetic photon flux density (PPFD) across treatments. McAusland et al. [11] conducted a detailed study on coriander (*Coriandrum sativum*) showing that light quality not only influences plant morphology and biomass production, but also significantly alters aroma profiles. Specifically, the concentration of the main compound in the essential oils of coriander grown under lighting conditions of R:B (1:1) and R:B:G (35.8:37.8:26.4) spectra is 3–4 times higher than that under lighting conditions of only R or only B spectrum. The enhanced aromatic complexity obtained under more diverse light spectra is believed to result from increased production of beneficial secondary metabolites associated with plant defense, competition, and overall fitness.

The addition of Fr to R:B spectral lights increasing the biomass of lettuce (*Lactuca sativa*) by 39% compared with the one under the treatment of R:B alone was reported [12]. Similarly, supplemental R:B and R:B:Fr lighting increased sweet pepper (*Capsicum annuum*) yields by 22 and 33%, respectively, compared with the one under natural light, whereas, R:B:Fr lighting increased red and yellow fruit yields by 9 and 19%, respectively, compared with the one under control treatment [13].

*Ocimum gratissimum* L. (OG), a member of the Lamiaceae family, is widely used as a natural flavoring agent, culinary spice, and leafy vegetable [14]. In agriculture, OG is considered a promising plant for repelling aphids, owing to its antifeedant or irritant effect on these pests [15]. In traditional medicine, OG has been employed to treat a variety of ailments, including colds, body aches, pneumonia, diarrhea, anemia, and bacterial and fungal infections [16]. Recent studies have demonstrated the neuroprotective effects of OG. For instance, Udi et al. [17] reported that Wistar rats exposed to 120 mg/kg of lead acetate and subsequently treated with OG at doses of 125 and 250 mg/kg for 21 days exhibited significant improvements in brain antioxidant markers, including glutathione (GSH), superoxide dismutase (SOD), and catalase (CAT). These findings suggest that OG mitigates cerebellar damage by reducing lipid peroxidation, boosting antioxidant defenses, and preserving the structural integrity of Purkinje cells.

In addition, the essential oil derived from OG leaves has shown inhibitory activity against PIM-1 kinase in *Escherichia coli*, primarily attributed to *α*-selinene—a key phytochemical component. Molecular docking studies revealed that α-selinene has a binding affinity of –7.8 kcal/mol, closely comparable to Apalutamide (–7.9 kcal/mol), a drug used in the treatment of prostate cancer. Based on these findings, regular consumption of OG leaves has been suggested as a potential dietary approach to reducing prostate cancer risk in middle-aged men [18]. Eugenol in OG leaf and inflorescence extracts shows a strong affinity for surface lysine residues and effectively inhibits advanced glycation end-product (AGE) formation. In diabetic mice, eugenol reduced blood glucose by 38%, likely via α-glucosidase inhibition, without affecting insulin or HbA1c levels. These findings suggest a dual antidiabetic mechanism: blocking glycation and lowering glucose, supporting its potential in diabetes management [19]. In a recent in silico study, 30 phytochemicals from OG were predicted to target 136 diabetes-related proteins, including aldose reductase, DPP4, α-amylase, and α-glucosidase. Rutin demonstrated the strongest binding affinity to aldose reductase (–11 kcal/mol) by forming 17 intermolecular interactions, suggesting that OG may exert anti-diabetic effects via multi-target and multi-pathway mechanisms [20].

The growth and the organic compounds biosynthesized and accumulated in OG vary significantly under different environmental conditions [21]. Light condition plays a crucial role in the variations in plant quality, e.g., higher light intensity linked to increased essential oil yield due to greater leaf biomass [22]. LED technology offers a powerful approach to optimizing plant growth [9], regulating physiological processes, and enhancing secondary metabolite production in plants. With benefits such as longevity, compact size, high efficiency, low thermal radiation, dimming ability, variety of LED-spectra and flexibility in agricultural applications, LEDs allow for tailored light spectra to provide sufficient photosynthetic photon density in terms of energy and meet plants’ specific absorption needs. This enables precise control over photomorphogenesis, accelerates the reproductive cycle, and improves plant quality by stimulating secondary metabolite accumulation [23]. Recently, Weeplian et al. (2025) [24] investigated the effects of supplemental Fr (710–740 nm) and UV-A (360–370 nm) light during late growth stages (42–49 days after sowing) on OG. The authors used a 200 µmol·m^−2^·s^−1^ light intensity with 80% white (W) LEDs and 20% R LEDs (660 nm) under a 16 h photoperiod. The results showed that Fr supplementation increased plant height (25.55 cm), branch number (12.8), but decreased shoot (13.6 g) and root fresh weight (4.53 g), and leaf number (11.1). UV-A light, in contrast, improved shoot weight (32.53 g), plant height (38.4 cm), and leaf number (18.4), but reduced branch number (11.4). Both Fr and UV-A increased secondary metabolites, including total phenolic content (TPC), total flavonoid content (TF), and total anthocyanins (ACN), as well as antioxidant activity (AA). Fr significantly boosted TPC, TF, and AA in both leaves and roots, while UV-A enhanced TPC and ACN in leaves and AA. In the roots, UV-A increased TPC and TF, yielding the highest AA values.

Previous studies have underscored the pivotal role of light quality (light spectra composition and exposure duration) in promoting biomass accumulation and enhancing the concentration of main essential oil constituents in plants. These findings present a valuable opportunity: by precisely manipulating light spectra, growers can simultaneously optimize yield, flavor, aroma, and plant morphology—an approach deeply grounded in plant physiological principles. Given that plant species respond differently to light stimuli [25], targeted investigations are necessary. However, to our best knowledge, research on the use of supplemental multispectral LED lighting in OG cultivation is still scarce [24]. Furthermore, no studies have evaluated the effects of LED lighting on the content, chemical composition and antimicrobial activity of OG essential oils.

Artificial lighting is energy-intensive and contributes significantly to production costs. Therefore, improving lighting efficiency is critical for sustainable horticulture. Accurate evaluation of spectral efficiency is essential to guide such improvements. In this study, our primary objective is to explore the physiological and phytochemical responses of OG to various light spectra, providing foundational insights into the effects of light quality on plant growth and its essential oil composition. To this end, we developed complex LED lighting systems using R (660 nm) and B (440 nm) lights as primary components, supplemented with UV-A (365 nm), G (530 nm), and/or Fr (730 nm) wavelengths. These spectral treatments were applied at night under varying irradiance levels and exposure durations. While the plants benefited from natural sunlight during the day in a net house environment—offering a significant energy-saving advantage over fully artificial greenhouse systems. Here we investigate the effects of supplemental light quality (light spectrum composition), irradiance levels and irradiation time periods at nighttime on the growth, essential oil yield, chemical composition, and antimicrobial activity of OG grown in a net house in Vietnam. We hypothesize that radiations with different wavelengths and their irradiance ratios, photon densities, and light–dark cycle would significantly influence the plant’s growth and phytochemical profile—potentially creating trade-offs for growers. For instance, certain lighting conditions might enhance biomass production while diminishing the intensity of the characteristic aroma, or vice versa. However, if such trade-offs are minimal, the use of spectrum-specific LEDs could represent a valuable opportunity to optimize both yield and quality in OG cultivation.

To test this hypothesis, OG plants were grown under natural sunlight during the day and supplemented with multi-spectrum LED lighting at night. Biomass was assessed, and essential oil yield was determined via hydrodistillation. The chemical composition of the oils was analyzed using gas chromatography–mass spectrometry (GC-MS) and gas chromatography–flame ionization detection (GC-FID), with a focus on identifying the major compounds responsible for OG’s characteristic aroma. Additionally, antimicrobial activity of the essential oils was evaluated using the microdilution method to determine any variations linked to supplemental lighting conditions. Our findings will suggest that light spectra significantly influence biomass production, essential oil yield, and the concentration of desirable aromatic compounds—highlighting the potential of LED lighting as a tool to manipulate and improve yield and quality in this useful medicinal plant. To our knowledge, this is the first study to evaluate the effects of supplemental multispectral LED lighting on OG cultivated in a net house over a 15-week period.

## 2. Results and Discussion

### 2.1. The Effect of Light Spectra on Biomass and Essential Oil Yield of Ocimum gratissimum

Under various supplemental light conditions, *Ocimum gratissimum* L. (OG) exhibited noticeable variations in growth parameters, biomass, and essential oil yield (Table 1). At the initiation of supplemental lighting, the average plant height was 15.15 ± 0.43 cm/plant. After 15 weeks of supplemental light treatments, OG heights varied across the 10 lighting regimens, although the differences were not statistically significant (*p* = 0.129) (Table 1). In general, all multi-spectral LED lighting treatments promoted greater plant height compared with the control (F10), which received only natural sunlight (97.63 ± 1.28 cm/plant). Specifically, treatments incorporating the red: blue: ultraviolet-A (R:B:UV-A) spectra (F1, F2, and F3) yielded the tallest plants (104.42 ± 1.51—117.55 ± 6.07 cm/plant. The significant elongation of plant height observed under the R:B:UV-A spectra aligns with previous findings in basil (*Ocimum basilicum*), where UV-A light notably increased plant height [26]. The red/blue/green (R:B:G) treatments also contributed to height increase (F4, F5, and F6; between 98.81 ± 9.25 and 104.45 ± 13.31 cm/plant). It is likely due to G light’s capacity to penetrate deeper into leaf tissues—reaching either the surface or middle mesophyll layers depending on intensity—thereby influencing photosynthetic activity and chlorophyll distribution [27]. Meanwhile, the integration of Fr light into the R:B:G spectra may have contributed to the increased plant height observed in OG (F7, F8, and F9; between 101.42 ± 6.75 and 102.51 ± 8.04 cm/plant). This result is similar to the effect on grapevine of Fr light, by enhancing photosynthetic efficiency [28]. Among the two spectral groups (R:B:UV-A and R:B:G), the 6 h daily supplemental lighting duration (F2 and F5), delivering a DLI of approximately 2.16 mol·m^−2^·d^−1^, was optimal for promoting stem elongation in OG. Both shorter (4 h/night) and longer (8 h/night) durations were less effective, likely due to disruption of the plant’s activity and rest regime. These findings are consistent with previous studies in basil (*Ocimum basilicum*), which indicated that plant needs a dark cycle for normal growth. Specifically, when basil was grown for 15 days under 24 h continuous light intensity, it showed signs of stunted growth, chlorosis, and leaf necrosis [29]. Similarly, an optimal photoperiod of 16–18 h/day was suggested for lettuce (*Lactuca sativa*) growth [30]. In contrast, variations in light intensity (80–120 µmol·m^−2^·s^−1^) under the R:B:G:Fr spectral group (F7, F8 and F9) did not produce notable differences in plant height, suggesting that within this range, light quality may play a more crucial role than quantity for this spectral treatment (Table 1).

Consistent with the trend observed for plant height, the aboveground fresh weight of supplemental LED lighting OGs significantly increased (*p* < 0.001) compared with the control (F10) (Table 1). The increase in plant biomass observed across the nine supplemental LED light treatments can be primarily attributed to the stimulating effects of R and B light spectra, as reported in previous studies on some other plants species [31,32]. Additionally, the integration of UV-A, G, and Fr lights in this study also contributed significantly to enhancing biomass production in OG. Specifically, among all spectral groups tested, treatments combining R, B, and UV-A light (F1, F2, and F3) resulted in the highest fresh biomass accumulation. Notably, the F2 treatment, which applied supplemental lighting for 6 h/day, resulted in the greatest fresh weight at 335.02 ± 18.25 g/plant (equivalent to 13.07 ± 0.71 Mg/ha). This value is nearly double that of the control with 161.40 ±1.22 g/plant (6.29 ± 0.05 Mg/ha). The fresh weight of OG from F2 treatment is also significantly higher (*p* < 0.001) than those from both F1 and F3. These results reflect a 62.16–107.79% increase in fresh biomass of OG in the R:B:UV-A spectral treatments over the control treatment. Recent study [24] also showed that UV-A when supplemented in combination with W and R lights resulted in an increase in height and fresh shoot biomass of OG. The increase in biomass under UV-A radiation has been associated with enhanced leaf chlorophyll content and improved photosynthetic performance in several plant species [33]. In the R:B:G group, the F4 treatment (8 h/night) produced the highest fresh weight at 227.07 ± 2.66 g/plant (8.86 ± 0.10 Mg/ha), which was significantly greater (*p* < 0.001) than both F5 and F6. For the R:B:G:Fr group, the F9 treatment (80 µmol·m^−2^·s^−1^) yielded the highest fresh biomass at 209.38 ± 3.50 g/plant (8.17 ± 0.14 Mg/ha), significantly surpassing (*p* < 0.001) F7 and F8.

Plant water content also varied significantly across lighting treatments. The highest water contents were observed in F5 (79.87 ± 0.21%) and F6 (79.82 ± 0.17%). In contrast, other treatments showed significant reductions (*p* < 0.001). These differences influenced the ranking of dry biomass yields, which did not follow the same order as fresh weights. Nonetheless, the R:B:UV-A group still consistently delivered the highest dry biomass yields among all treatments. The superior performance of the R:B:UV-A spectrum agrees with previous reports showing that UV-A, when combined with R and B lights, enhances basil (*Ocimum basilicum*) biomass [23], as well as increases plant height, leaf area, and leaf mass [26]. Similar growth-promoting effects of UV-A were also observed in lettuce (*Lactuca sativa*), with increases in shoot biomass, leaf area, and leaf number [5]. G light was also reported to stimulate plant growth due to its capacity to penetrate deeper into plant tissues and affect inner mesophyll cells [27]. Likewise, the combination of Fr and R light was shown to increase plant height and stem biomass in *Capsicum annuum* compared with R light alone [34]. In addition, Fr supplementation was reported to significantly enhance both plant height and dry weight in foxglove (*Digitalis purpurea*) by up to 38% [35]. This light wavelength was also shown to increase dry weight of lettuce (*Lactuca sativa*) by 46–77% depending on planting densities [36].

The essential oil concentration of OG was significantly affected by supplemental lighting conditions (*p* < 0.001), ranging from 0.80 ± 0.002% to 1.12 ± 0.002% (*v*/*w*), calculated on a dry weight (DW) basis, all higher than the control formula F10 (0.76 ± 0.001%, *v*/*w*) (Table 1). These values also exceed the previously reported value of 0.7% [37]. The highest essential oil concentration was observed in treatment F2 (1.12 ± 0.002%), which corresponded with its superior plant height and fresh biomass. This was followed by F7 and F6. Notably, in the R:B:G:Fr spectral group, higher light intensity (F7 at 120 µmol·m^−2^·s^−1^) led to greater oil concentration (1.03 ± 0.001%) compared with the one (0.81 ± 0.001 and 0.81 ± 0.002%) in lower intensities (F8 and F9 at 100 and 80 µmol·m^−2^·s^−1^). This finding contrasts with a previous study [22], which reported no significant impact of light radiation on OG oil concentration. However, the effect of light on essential oil accumulation is known to vary by species and spectral quality. For instance, R and Fr light supplementation was shown to enhance essential oil concentrations in cilantro (*Coriandrum sativum*), dill (*Anethum graveolens*), and parsley (*Petroselinum crispum*) [38], as well as in rosemary (*Rosmarinus officinalis*—now it is a synonym of *Salvia rosmarinus*) [39]. In the current study, the essential oil yield of OG, calculated based on data of dry biomass and essential oil concentration, ranged from 12.08 ± 0.09 to 31.39 ± 1.71 L/ha. Consistent with the trends in plant height and biomass, treatments under the R:B:UV-A spectral group produced significantly higher oil yields (20.79 ± 0.06–31.39 ± 1.71 L/ha) compared with both other LED spectral combinations and the control treatment. This corresponds to an increase in essential oil yields of OG under the R:B:UV-A spectral treatments, ranging from 72.10 to 159.85% compared with the control. Among these treatments, the F2 achieved the highest essential oil yield at 31.39 ± 1.71 L/ha, confirming it as the most effective supplemental lighting condition for maximizing both biomass and essential oil production of OG.

Overall, the three spectral lighting systems (R:B:UV-A, R:B:G and R:B:G:Fr) comprising nine supplemental light formulas significantly increased the fresh biomass and essential oil yield of OG compared with the control treatment receiving only natural sunlight. Among these, the spectral group containing UV-A light (F1, F2, and F3) demonstrated the most pronounced effects. These results highlight the superior efficacy of the R:B:UV-A spectral combination in promoting OG growth and secondary metabolite production compared with other tested light spectra. This finding aligns with previous studies reporting that R, B, and UV light can significantly enhance essential oil biosynthesis across various plant species [2], while G light was shown to exert a comparatively lower influence on biomass accumulation [40].

### 2.2. The Effect of Light Spectra on Essential Oil Composition of Ocimum gratissimum

The chemical composition and physical properties of OG essential oil are crucial indicators of plant quality, complementing oil yield in overall evaluation. The aerial parts of OG plants harvested from the ten different lighting treatments were shredded and subjected to hydrodistillation, yielding pale yellow essential oils. The physical characteristics of the oils varied slightly among treatments, with relative densities (*d*^20^) ranging from 0.9785 to 0.9984 g/mL, refractive indices (*n*^20^) from 1.5138 to 1.5190, and optical rotations [α]D^20^ from [−]27.78 to [−]16.53° (Table 2). Notably, the measured relative densities were slightly lower than those previously reported for OG essential oil [37].

The OG essential oils contained 21–28 quantified compounds, accounting for 97.8 to 99.4% of the total composition. Phenylpropanoids (59.4–71.2%) were the predominant class, followed by sesquiterpene hydrocarbons (12.3–21.3%) and monoterpene hydrocarbons (11.8–14.6%) (Table 3).

**Figure 1:**
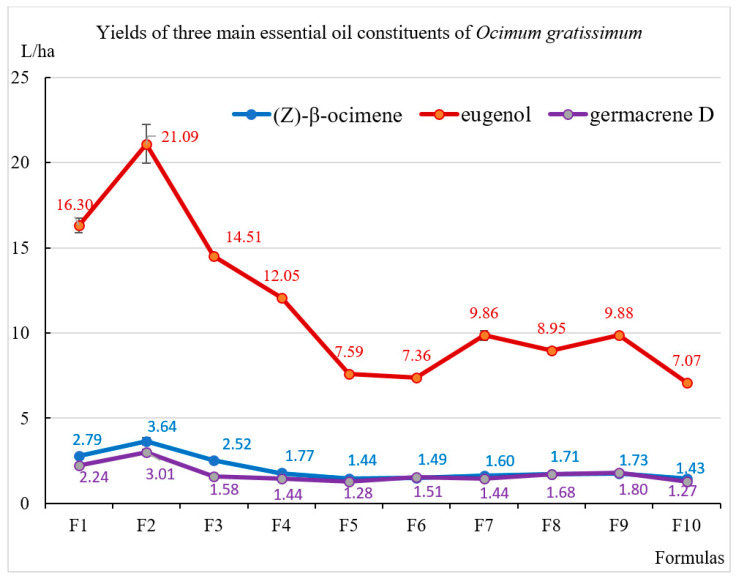
The yields of the three major compounds in essential oils of *Ocimum gratissimum* cultivated under different light conditions.

Significant variations were observed among the ten OG essential oils, particularly in the concentrations of three major constituents: (i) (*Z*)-*β*-ocimene (10.2–12.1%), (ii) eugenol (58.5–69.8%), and (iii) germacrene D (7.6–12.1%). In general, OG plants subjected to supplemental lighting treatments (F1–F9) exhibited higher eugenol concentrations than those grown under natural sunlight alone in formula F10 (Figure 1). Among these, the spectral group incorporating R:B:UV-A (F1, F2, and F3) had the most pronounced effect in enhancing eugenol biosynthesis and accumulation, followed by the R:B:G group, and lastly the R:B:G:Fr group. Notably, the highest eugenol and (*Z*)-*β*-ocimene concentrations were recorded under the F3 treatment (71% R: 20% B: 9% UV-A at 100 µmol·m^−2^·s^−1^ for 4 h/day). In contrast, germacrene D peaked in F6 and was lowest in F3. These results suggest that UV-A light, when combined with R & B spectra, is particularly effective in promoting eugenol synthesis in OG, more so than the combinations involving G and Fr lights. The increased eugenol concentration in OG essential oil under supplemental UV-A illumination may be due to the strong activation of phenylalanine ammonia-lyase (PAL) and chalcone synthase (CHS) genes (the first steps in the phenylpropanoid pathway) as in the case of basil (*Ocimum basilicum*) [41]. PAL and CHS play a fundamental role in energy production and precursors for later enzymes such as eugenol synthase (EGS), which is an important prerequisite for increased eugenol synthesis.

Within the R:B:G:Fr spectral group, a higher light intensity in F7 (at DLI of 16.57 mol·m^−2^·d^−1^) also increased eugenol concentration compared with lower intensities in F8 and F9 (at DLI of 16.14 and 15.71 mol·m^−2^·d^−1^, respectively). This finding is different from the previous results [22], which reported no significant effect of sunlight intensity (4, 7, 11, and 20 mol·m^−2^·d^−1^) on essential oil composition, possibly due to the unaltered density of glandular trichomes. Another compound present in relatively high amounts was (*E*)-*β*-caryophyllene (3.3–6.9%). The remaining compounds were detected at much lower concentrations, generally from trace levels up to 1.7%. Overall, when R and B were used as the primary light sources, changes in the metabolite levels of OG—particularly eugenol, the major constituent of its essential oils in this study—were significant only under certain treatments, depending on the integrated spectral components and light dosage. The substantial fluctuations in secondary metabolite concentrations were also reported in plants exposed to different monochromatic light conditions. For instance, in certain Lamiaceae species such as sweet mint (*Mentha spicata*) [42], lemon balm (*Melissa officinalis*) [32], and sweet basil (*Ocimum basilicum*) [43], essential oil composition varied distinctly under R, B, and/or G light treatments. However, contrasting results were also observed. Aghakarim et al. [44] reported no significant differences in the concentrations of the three main essential oil constituents of lemon balm under R and B light conditions. In another study, the combination of R or B light with W light reduced the concentration of menthol—the main compound in mint (*Mentha arvensis*) essential oil—compared to plants grown under W light alone [45].

The chemotype of essential oil extracted from the aboveground parts of OG in this study—characterized by the dominance of eugenol, (*Z*)-*β*-ocimene, and germacrene D—is consistent with that reported for OG leaves in previous studies [46]. However, the concentration of eugenol observed here differs from earlier findings, such as those by Freire et al. [47], who reported seasonal variations in eugenol concentration ranging from 44.89 to 56.10%, and Joshi [46], who noted a broader range from 65.65 to 85.71%. The predominance of eugenol in OG essential oil is in agreement with several other studies. For instance, Matasyoh et al. [48] reported eugenol (68.8%), methyl eugenol (13.21%), and (*Z*)-*β*-ocimene (7.47%) in OG grown in Kenya. Similarly, essential oils from Algeria contained eugenol (54.8%) and β-elemene (10.9%) [49]; while those from India contained eugenol (75.1%) and terpinolene (14.2%) [50], or eugenol (57.1%), α-bulnesene (15.6%), and (*E*)-β-caryophyllene (14.2%) [51]. Nonetheless, other studies have identified different dominant constituents in OG essential oils, likely due to variations in environmental conditions such as soil composition, geographic location, and seasonal factors. For example, Martins et al. [52] reported *p*-cymene (12.5%) and thymol (48.1%) as the major components of samples in Portugal, while Coulibaly et al. [53] identified *p*-cymene (12.9%), γ-terpinene (20.5%), and thymol (29.5%) in samples from Burkina Faso.

In general, yields of the three major constituents in essential oils of OG were markedly higher under R:B:UV-A supplemental lighting compared with other treatments. Notably, the highest yields were recorded in treatments F2 and F1. Each of these compounds possesses significant bioactive properties. Eugenol is a well-known natural compound with broad applications in food, aromatherapy, cosmetics, agriculture, and pharmaceuticals due to its diverse pharmacological effects across various biological systems [54]. In particular, eugenol demonstrates notable anticancer activity by modulating several key biological pathways, including apoptosis, autophagy, cell cycle regulation, inflammation, invasion, and metastasis [55]. In addition, it exhibits protective effects against arthritic inflammation, respiratory pathologies, and oxidative liver damage. Eugenol also plays a vital role in regulating systemic inflammatory responses, enhancing defense against pathogenic microorganisms, and exerting neuroprotective and antidiabetic effects [56]. In another study, eugenol has been reported to exhibit neuroprotective effects in polyglutamine-induced degeneration in *Drosophila* model [57]. Moreover, eugenol strongly inhibits biofilm and quorum sensing and reduces the expression of bacterial virulence factors [58]. (*Z*)-*β*-Ocimene, a widely occurring monoterpene, was reported to be associated with anticonvulsant, antifungal, antitumor, and insect-repellent activities [59]. Meanwhile, germacrene D is a common volatile sesquiterpene that serves as a biogenetic precursor to several other sesquiterpene derivatives, including muurolane, cadinane, and amorphane types [60].

### 2.3. The Effect of Light Spectra on Antimicrobial Activity of Essential Oils of O. gratissimum

The antimicrobial activity of OG essential oil was evaluated against three strains of Gram-positive bacteria (*Staphylococcus aureus*, *Bacillus subtilis*, *Lactobacillus fermentum*), three strains of Gram-negative bacteria (*Salmonella enterica*, *Escherichia coli*, *Pseudomonas aeruginosa*), and one strain of yeast (*Candida albicans*). The oil samples showed weak inhibitory effects against *C. albicans*, with IC_50_ and MIC values ranging from 2450 to 3750 µg/mL and from 4096 to 16,384 µg/mL, respectively. Spectral group containing R:B:G:Fr (F7, F8, and F9) exhibited higher activity against *C. albicans* compared with other treatments. Notably, only the essential oils of OG cultivated under condition F1 demonstrated inhibition against *E. coli*, with IC_50_ and MIC values of 6051 µg/mL and 16,384 µg/mL, respectively. For the remaining five tested microorganisms, the IC_50_ and MIC values of the oils exceeded 16,384 µg/mL (Table 4).

The antimicrobial activity of OG oil is likely due to the synergistic effect of eugenol and other constituents present in the essential oil [50]. Previous studies showed that the essential oil from the aerial parts of OG and/or eugenol exhibits activity against various phytopathogenic fungal strains [61], dermatophyte fungal strains [62], and some microbial and yeast strains [48]. The lethal mechanism of eugenol on fungi involves either cell cycle arrest or disruption of fungal cell membrane integrity [63]. Due to its lipophilic nature, eugenol accumulates in the phospholipid bilayer of fungal cells, altering the function of important membrane-bound enzymes and affecting membrane permeability, fluidity, and morphology [64,65]. Due to its notable anti-*Candida* activity, eugenol has been investigated as a potential adjunct to antifungal therapy. For instance, when combined with fluconazole or itraconazole, eugenol enhances the synergistic effect against *Candida albicans* by disrupting biofilm structure and increasing drug permeability [66].

While the antifungal activity of OG essential oil grown under treatments supplemented with light from the R:B:G:Fr spectral group was higher than in other treatments, this study did not reveal a clear relationship between supplemental lighting conditions and the overall antimicrobial activity of the oil. However, supplemental lighting using different light spectra can influence the concentration of the main compounds in OG oil. Future studies may identify a specific light spectrum or combinations of spectra that significantly alter the concentrations of these key compounds, potentially enhancing the antimicrobial activity of the oil.

Thus, all supplemental multispectral LED treatments (F1–F9) enhanced fresh biomass, essential oil concentration, and the major essential oil constituent of OG compared with the control (F10), which received only sunlight. Notably, treatments F5, F6, and F7 produced lower dry biomass than the control, although their essential oil yields remained higher. This supports our initial hypothesis of a potential trade-off. Under these three treatments, OG plants accumulated higher fresh biomass than the control, but their higher water content resulted in reduced dry matter. By contrast, the remaining six LED treatments consistently promoted OG growth, essential oil biosynthesis and accumulation beyond the control. Among these, treatment F2 emerged as the optimal supplemental multispectral light condition for OG cultivation.

Similar trade-offs have been reported in other essential oil–bearing plants. For instance, *Thymus vulgaris* grown under multispectral LEDs showed increased fresh biomass, dry biomass, and volatile compound content compared with other lighting conditions. However, the leaf-to-shoot ratio declined, indicating that although overall biomass increased, the relative proportion of foliage—and thus foliage quality—was potentially reduced [67]. Likewise, *Coriandrum sativum* exposed to high-intensity continuous LED lighting (400 µmol·m^−2^·s^−1^, 24 h) achieved greater yields but at the cost of reduced photosynthetic efficiency and energy use efficiency [68].

## 3. Materials and Methods

### 3.1. Plant Materials, Growth Conditions and Light Treatments

*Ocimum gratissimum* L. (OG) seeds were purchased in Hanoi and sown on 2 April 2024. After 7 weeks, when the seedlings reached approximately 15 cm in height, uniform plants were selected for transplantation into experimental plots within the net house. The plants were arranged at a density of 40 × 50 cm. The experiment followed a completely randomized design with three replications per treatment, totaling 30 plots, each containing 15 plants. Nutritional inputs, irrigation, and natural sunlight exposure were maintained uniformly across all plots. After a one-month stabilization period, in June, the supplemental lighting treatments were initiated following the formulas listed in Table 5 to assess their effect on OG growth and essential oil accumulation. At the start of the supplemental lighting phase, the average plant height was 15.15 ± 0.43 cm/plant. After 15 weeks of treatment with supplemental LED irradiation at nighttime, in September, the entire aboveground biomass of OG was harvested for further analysis. The plant sample was identified by Assoc. Prof. Dr. Hai Van Do, a plant expert at the Department of Botany, Institute of Biology, Vietnam Academy of Science and Technology. Voucher (code: HN 000080501) was deposited at the herbarium HN of VAST.

The experiments were conducted in 2024 in a net house in Hanoi, Vietnam (N 21°04′08″, E 105°45′50″), with 40% diffused light transmission, determined by comparing the photosynthetic photon flux density (PPFD) (400–700 nm) inside and outside the net house, using a LI-250A Light Meter (Biosciences, Torrance, CA, USA) equipped with a LI-190SA Quantum Sensor and a 2003S Mounting and Leveling Fixture (Biosciences, USA). The average PPFD during the experiment was 13.98 mol·m^−2^·d^−1^, with monthly averaged values recorded as 15.02 in June, 14.20 in July, 14.03 in August, and 12.64 in September, based on the daily light integral (DLI) database from SuntrackerTech Technologies Ltd.; British Columbia, Canada [69]. To investigate the effects of spectral composition on plant physiological responses and secondary metabolite production, three distinct supplemental light-emitting diode (LED) spectral groups were selected based on established principles of plant photobiology. These spectral lights were shown to be highly effective in several herbal plants [3,9,11,13]. The first spectrum group consisting of red/blue/ultraviolet-A (R:B:UV-A) at the ratio of 71:20:9 was formulated to stimulate the biosynthesis of secondary metabolites. The second spectrum group consisting of red/blue/green (R:B:G) at the ratio of 75:21:4 targeted plant growth by incorporating G light into a high R-to-B ratio to help light deeper penetration and more uniform photon distribution within the canopy. The third spectrum group consisting of red/blue/green/far-red (R:B:G:Fr) at the ratio of 43.5:43.5:8:5 was designed to approximate the natural solar spectrum by balancing R and B lights while integrating G and Fr wavelengths to influence phytochrome-mediated developmental processes in plants. Three variations in light intensities (80, 100, and 120 µmol·m^−2^·s^−1^) and photoperiod durations (4, 6, and 8 h per night) were used to assess dose-dependent OG’s responses. The supplemental lighting was applied both before sunrise (from 1.00 or 2.00 or 3.00 to 5.00 a.m.) and after sunset (from 7.00 to 9.00 or 10.00 or 11 p.m.) to extend the photoperiod without overlapping with natural daylight. The lighting regimen was maintained for 15 weeks, from June to September, covering the period from the stabilization phase to flowering. An irradiance of 80–120 µmol·m^−2^·s^−1^ was applied at the plant canopy, with the LED lamps positioned approximately 50 cm above the plants. Ptak et al. (2021) [70] reported that at this distance the plants received sufficient irradiance to elicit physiological responses without causing photo damage or excessive heat stress. A control formula without supplemental lighting was included, bringing the total number of treatments to ten with DLI values measured ranging from 13.98 to 16.86 mol·m^−2^·d^−1^ (Table 5). The LED lights emitted a broad continuous spectrum, including UV-A (365 nm), B (440 nm), G (530 nm), R (660 nm), and Fr (730 nm), as measured by a USB2000+ Fiber Optic Spectrometer (Ocean Optics, Orlando, FL, USA).

### 3.2. Essential Oil Extraction and Physical Properties Analysis

Each plant sample was measured for water content using A&D Weighing AD-4714A General purpose moisture determination balance (A&D Company, Limited, Tokyo, Japan) at 105 °C for 35 min. Fresh biomass sample of OG (1.5 kg each including stems, leaves, and flowers) was shredded and subjected to hydrodistillation in triplicate for four hours using a Clevenger-type apparatus following the standard procedure outlined by the Ministry of Health of Vietnam [71]. The plant sample was placed in the distillation pot, after which 1.3 L of tap water were added, and the pot was sealed tightly with its lid. A glass condenser tube was connected to the hole on the lid using a flexible rubber gasket to ensure an airtight system. Cooling water was continuously supplied through the condenser by opening the faucet valve. The mixture was then heated on an electric stove, and after approximately 20 min, steam began to condense, yielding a distillate composed of essential oil and water. To ensure efficient condensation, the stove temperature was maintained at a moderate level, as excessive heating could generate excess steam that might escape through the condenser vent as vapor. Because most essential oils are less dense than water, they separated naturally from the aqueous phase and accumulated on the surface of the distillate. Meanwhile, the condensed water was re-circulated back into the distillation pot through the diagonal tube of the condenser system. The oil concentrations were calculated on the dry weights of OG based on the measured water contents. The extracted essential oil was separated and stored at −5 °C for further analysis. Three physical properties of OG essential oils consisting of relative density, refractive index, and optical rotation, were evaluated according to the methods given in ISO standards [72,73,74].

### 3.3. Gas Chromatography/Mass Spectrometry with Flame Ionization Detection Analysis

The chemical composition of the essential oils was analyzed using Gas Chromatography/Mass Spectrometry with Flame Ionization Detection (GC/MS-FID) on an Agilent GC7890A system equipped with a Mass Selective Detector (Agilent 5975C, Agilent Technologies, Santa Clara, CA, USA). Separation was achieved using an HP-5MS fused silica capillary column (60 m × 0.25 mm i.d. × 0.25 μm film thickness), with helium as the carrier gas at a flow rate of 1.0 mL/min. The inlet temperature was maintained at 250 °C, while the oven temperature was programmed to increase from 60 °C to 240 °C at a rate of 4 °C/min. The split ratio was 100:1, and the detector temperature was maintained at 280 °C, with an injection volume of 1 μL. For mass spectrometry, the interface temperature was 280 °C using an E.I. detector voltage of 70 eV. Mass spectra were recorded in the range of 35–450 Da at a scan rate of 4.0 scans/s. The GC-FID analysis was performed under identical chromatographic conditions, with the FID temperature set at 250 °C. Essential oil components were identified based on their relative retention indices, determined through co-injection with a series of homologous *n*-alkanes (C7–C30), and by comparing their mass spectral fragmentation patterns with those stored in the MS library NIST08, Wiley09, and HPCH1607 [75,76]. Data processing was performed using MassFinder 4.0 software [77], and the relative concentrations of components were calculated based on peak areas from FID chromatograms without standardization.

### 3.4. Antimicrobial Activity Screening

The antimicrobial activities of the OG oils were evaluated against three Gram (+) bacterial strains—*Staphylococcus aureus* (ATCC 13709), *Bacillus subtilis* (ATCC 6633) and *Lactobacillus fermentum* (VTCC N4), and three Gram (–) bacterial strains—*Salmonella enterica* (VTCC), *Escherichia coli* (ATCC 25922) and *Pseudomonas aeruginosa* (ATCC 15442), as well as one yeast strain—*Candida albicans* (ATCC 10231). The ATCC strains were sourced from the American Type Culture Collection, while the VTCC strains were obtained from the Vietnam Type Culture Collection at the Institute of Microbiology and Biotechnology, Vietnam National University, Ha Noi.

The minimum inhibitory concentration (MIC) and half-maximal inhibitory concentration (IC_50_) values of the essential oils were determined in triplicate using the broth microdilution susceptibility testing [78,79]. Stock solutions of the oil were prepared in dimethylsulfoxide (DMSO), and serial dilutions ranging from 16,384 to 2 μg/mL (10 concentrations including: 2^14^, 2^13^, 2^12^, 2^11^, 2^10^, 2^9^, 2^7^, 2^5^, 2^3^, 2^1^ μg/mL) were prepared in sterile distilled water in micro-test tubes, which were then transferred to 96-well microplates. Bacterial cultures were grown in double-strength Mueller-Hinton broth or double-strength tryptic soy broth, while fungal cultures were grown in double-strength Sabouraud dextrose broth. Microbial suspensions were standardized to 5 × 10^5^ for bacteria and 1 × 10^3^ CFU/mL for fungi. Wells containing only the culture medium without the serial dilutions of the essential oil and without microorganisms served as a negative control. While culture medium containing microorganisms without the serial dilutions of the essential oil were used as a positive control. Following incubation at 37 °C for 24 h, MIC values were determined at the well with the lowest concentration of agents at which microbial growth was completely inhibited. IC_50_ values were calculated based on microbial growth inhibition percentages, using turbidity measurements obtained from an EPOCH2C spectrophotometer (BioTeK Instruments, Winooski, VT, USA) and analyzed with Rawdata computer software, 2005 (Intercity Business Park Mechelen Noord, Mechelen, Belgium) according to the following equations:

(1) $$\%\;inhibition=\frac{{OD}_{control\left(+\right)}-\;{OD}_{test\;agent}}{{OD}_{control\left(+\right)}-{OD}_{control\left(-\right)}}\times 100\%$$

(2) $${IC}_{50}={High}_{Conc\;}-\frac{{(High}_{Inh\%\;}-50\%)\;{(High}_{Conc}-{Low}_{Conc})}{\left({High}_{Inh\%}-{Low}_{Inh\%}\right)}$$

where *OD* (optical density): Used to quantify microbial growth in the presence or absence of test agents; *control*(+): Culture medium containing microbial cells without any antimicrobial agent; *test agent*: Culture medium containing microbial cells and a known concentration of the test antimicrobial agent; *control*(–): Culture medium without microbial cells (blank control). *High_Conc_*/*Low_Conc_*: High and low concentrations of the test antimicrobial agent used in the assay; *High_Inh%_*/*Low_Inh%_*: Percent inhibition of microbial growth at high and low concentrations of the test agent, respectively.

Reference materials: Ampicillin for Gram (+) bacteria: IC_50_ range = 0.02–3.62 µg/mL; MIC range = 0.125–32.0 µg/mL, cefotaxime for Gram (–) bacteria: IC_50_ range = 0.07–4.34 µg/mL; MIC range = 0.5–32.0 µg/mL, nystatin for fungal strain: IC_50_ = 1.32 µg/mL; MIC = 8.0 µg/mL.

### 3.5. Statistical Analysis

Data on physiological characteristics and essential oil parameters of OG were analyzed using a single factor completely randomized analysis of variance (ANOVA) to compare the effect of different supplemental lighting treatments. Significance differences among means were determined using the least significant difference (LSD) test at a confidence level of *p* ≤ 0.05. All statistical analyses were performed using IRRISTAT ver. 5.0 (International Rice Research Institute, Laguna, Philippines).

## 4. Conclusions—Outlook

All supplemental multispectral LED lighting treatments conducted at nighttime positively influenced OG growth compared with that of the control under natural sunlight alone. After 15 weeks, these plants exhibited increased height, fresh aboveground biomass, essential oil concentration, essential oil yield, and eugenol concentration. Among those, the spectral group containing R:B:UV-A produced the most remarkable positive effects. These results suggest that supplemental R:B:UV-A light treatments could be an effective method to enhance phytochemical biosynthesis in plants, offering substantial commercial benefits. Especially, formula F2 (71% R, 20% B, 9% UV-A at 100 µmol·m^−2^·s^−1^ for 6 h/day, with a total daily light of 16.14 mol·m^−2^·d^−1^) was identified as the most optimal for promoting OG growth, biosynthesis, and essential oil accumulation. Although, no clear relationship was found between supplemental lighting conditions and the antimicrobial activity of the oil, the LED treatments significantly enhanced biomass and essential oil yield of OG. This result aligns with our expectation of identifying the optimal supplemental lighting conditions for OG growth and essential oil accumulation. These findings suggest promising potential for optimizing both plant yield and quality through specific light treatments. The research results provide both scientific and practical foundations for enhancing the growth and productivity of OG and other medicinal plants through the application of supplemental multi-spectral LED lighting. This approach can be especially beneficial for cultivating plants in diverse geographical, climatic, and seasonal conditions.

The authors of this paper recognize that evaluating the economic feasibility of supplemental multispectral LED lighting in the nighttime in addition to the main illumination with natural light in the daytime for OG cultivation is an important consideration from the technological and economic point of view. However, the primary objective of our study presented herewith was to investigate the physiological and phytochemical responses of the plant to different light spectra, providing foundational insights into the effects of light quality on its growth and essential oil composition. While a detailed cost–benefit analysis is beyond the scope of this study, we acknowledge its relevance for practical implementation and suggest that future studies address the economic viability of applying multispectral LED lighting in commercial-scale OG production. In several industry nations, the electricity costs may be expensive so that utilization of the natural light in the daytime and application of the supplemental LED lighting with defined durations in the nighttime in which the electricity costs are often relatively lower will be meaningful.

## Figures and Tables

**Table 1:** Biomass and essential oil yield of *Ocimum gratissimum* cultivated under different light conditions.

Formulas	Plant Height (cm/Plant)	Fresh Biomass Yield (Mg/ha) *	Water Content (%)	Dry Biomass Yield (Mg/ha) *	Essential Oil Content (%, Dry *v*/*w*)	Essential Oil Yield (L/ha) *
F1	104.42 ± 1.51 ^b^	10.50 ± 0.28 ^b^	71.31 ± 0.14 ^i^	3.01 ± 0.08 ^a^	0.80 ± 0.002 ^i^	24.05 ± 0.64 ^b^
F2	117.55 ± 6.07 ^a^	13.07 ± 0.71 ^a^	78.63 ± 0.18 ^c^	2.79 ± 0.15 ^b^	1.12 ± 0.002 ^a^	31.39 ± 1.71 ^a^
F3	110.49 ± 9.62 ^a^	10.20 ± 0.03 ^c^	77.93 ± 0.15 ^d^	2.25 ± 0.01 ^c^	0.92 ± 0.001 ^d^	20.79 ± 0.06 ^c^
F4	101.29 ± 4.24 ^b^	8.86 ± 0.10 ^d^	76.30 ± 0.17 ^e^	2.10 ± 0.02 ^d^	0.83 ± 0.002 ^f^	17.34 ± 0.20 ^d^
F5	104.45 ± 13.31 ^b^	6.82 ± 0.08 ^f^	79.87 ± 0.21 ^a^	1.37 ± 0.02 ^i^	0.89 ± 0.002 ^e^	12.22 ± 0.15 ^g^
F6	98.81 ± 9.25 ^b^	6.37 ± 0.14 ^g^	79.82 ± 0.17 ^a^	1.29 ± 0.03 ^k^	0.97 ± 0.002 ^c^	12.52 ± 0.27 ^g^
F7	101.42 ± 6.75 ^b^	6.96 ± 0.19 ^f^	78.90 ± 0.19 ^b^	1.47 ± 0.04 ^h^	1.03 ± 0.001 ^b^	15.14 ± 0.42 ^f^
F8	101.55 ± 6.12 ^b^	6.98 ± 0.13 ^f^	73.20 ± 0.17 ^h^	1.87 ± 0.04 ^f^	0.81 ± 0.001 ^g^	15.17 ± 0.29 ^f^
F9	102.51 ± 8.04 ^b^	8.17 ± 0.14 ^e^	75.39 ± 0.16 ^f^	2.01 ± 0.03 ^e^	0.81 ± 0.002 ^h^	16.20 ± 0.27 ^e^
F10	97.63 ± 1.28 ^b^	6.29 ± 0.05 ^g^	74.60 ± 0.19 ^g^	1.60 ± 0.01 ^g^	0.76 ± 0.001 ^k^	12.08 ± 0.09 ^g^
5%LSD	12.9709	0.4661	0.3071	0.1049	0.0028	1.0927
*p*	0.129	<0.001	<0.001	<0.001	<0.001	<0.001

Note: Mean values followed by the same letter within a column are not statistically different for 0.05 significant level (*n* = 15 × 3 times replicated). Statistical analyses were performed using IRRISTAT ver. 5.0 (International Rice Research Institute, Laguna, Philippines). * Estimated values (cultivation distance of OG: 40 × 50 cm, estimated number: 39,000 plants per ha).

**Table 2:** Some physical properties of essential oils of *Ocimum gratissimum* cultivated under different light conditions.

Parameters	F1 *	F2 *	F3 *	F4 *	F5 *	F6 *	F7 *	F8 *	F9 *	F10 *
Relative density *d*^20^	0.9951	0.9873	0.9984	0.9910	0.9853	0.9811	0.9900	0.9785	0.9851	0.9788
Refractive index *n*^20^	1.5155	1.5165	1.5186	1.5190	1.5168	1.5150	1.5170	1.5148	1.5168	1.5138
Optical rotation [α] _D_^20^	[−]21.36	[−]21.52	[−]16.53	[−]18.92	[−]24.10	[−]27.78	[−]21.46	[−]26.83	[−]26.65	[−]24.78

Note: * Standard deviation was insignificant and excluded from the Table to avoid congestion (*n* = 3).

**Table 3:** Composition of essential oils (%) of *Ocimum gratissimum* cultivated under different light conditions.

Compounds ^a^	RI ^b^	F1 ^c^	F2 ^c^	F3 ^c^	F4 ^c^	F5 ^c^	F6 ^c^	F7 ^c^	F8 ^c^	F9 ^c^	F10 ^c^
(3*Z*)-Hexen-1-ol	850	0.4	0.5	0.8	0.6	0.4	0.4	0.4	0.4	0.2	0.5
α-Thujene	929	0.3	0.3	0.3	0.2	0.3	0.4	0.3	0.5	0.4	0.5
1-Octen-3-ol	976	0.1	0.2	0.2	0.2	0.2	0.2	0.1	0.2	0.1	0.3
Sabinene	978	0.3	0.3	0.3	0.3	0.4	0.4	0.4	0.5	0.5	0.5
Myrcene	991	0.3	0.3	0.3	0.2	0.4	0.4	0.3	0.4	0.4	0.5
α-Terpinene	1021	0.1	Tr	0.1	0.1	0.1	0.1	0.2	0.2	0.2	0.2
*p*-Cymene	1029	Tr	Tr	Tr	Tr	0.1	0.1	0.1	0.2	0.2	0.2
(*Z*)-β-Ocimene	1038	11.6	11.6	12.1	10.2	11.8	11.9	10.6	11.3	10.7	11.8
(*E*)-β-Ocimene	1048	0.5	0.5	0.5	0.4	0.5	0.4	0.4	0.4	0.4	0.4
γ-Terpinene	1063	0.2	0.2	0.3	0.2	0.3	0.2	0.3	0.3	0.3	0.3
*cis*-Sabinene hydrate	1072	0.2	0.2	Tr	0.1	0.2	0.4	0.3	0.4	0.4	0.2
Linalool	1101	0.2	0.2	0.2	0.2	0.2	0.3	0.2	0.3	0.3	0.3
*allo*-Ocimene	1131	0.2	0.2	0.2	0.2	0.2	0.2	0.2	0.2	0.2	0.2
Menthol	1179	Tr	Tr	Tr	0.1	0.2	0.2	0.2	0.2	0.1	0.2
*iso*-Menthol	1179	Tr	0.2	0.2	Tr	Tr	Tr	Tr	Tr	Tr	Tr
Santalone	1185	0.5	0.4	0.5	Tr	Tr	Tr	Tr	Tr	Tr	Tr
Terpinen-4-ol	1186	Tr	Tr	Tr	0.5	0.6	0.5	0.7	0.8	0.7	0.9
Methyl chavicol (=Estragole)	1204	1.0	0.9	1.1	1.7	1.1	1.1	0.9	0.9	0.8	0.9
Eugenol	1369	67.8	67.2	69.8	69.5	62.1	58.8	65.1	59.0	61.0	58.5
α-Copaene	1390	0.7	0.7	0.6	0.6	0.9	1.0	0.9	1.0	1.0	1.0
(*E*)-β-Caryophyllene	1438	4.4	4.5	3.4	4.0	5.6	6.6	5.7	6.8	6.5	6.9
β-Gurjunene (=Calarene)	1446	Tr	Tr	Tr	Tr	0.1	0.2	0.1	0.2	Tr	0.2
α-Humulene	1472	0.3	0.3	0.3	0.3	0.4	0.5	0.4	0.5	0.5	0.5
γ-Muurolene	1491	Tr	Tr	Tr	0.2	0.2	0.2	0.1	0.2	0.2	0.2
Germacrene D	1499	9.3	9.6	7.6	8.3	10.5	12.1	9.5	11.1	11.1	10.5
γ-Cadinene	1531	Tr	Tr	Tr	Tr	Tr	Tr	Tr	Tr	Tr	0.2
δ-Cadinene	1537	0.4	0.4	0.4	0.4	0.6	0.7	0.6	0.7	0.6	0.7
Caryophyllene oxide	1605	0.3	0.3	0.2	0.4	0.5	0.5	0.3	0.6	0.5	0.5
*epi*-α-Cadinol (=τ-Cadinol)	1659	Tr	Tr	Tr	0.2	0.3	Tr	Tr	0.3	Tr	Tr
*epi*-α-Muurolol (=τ-Muurolol)	1660	Tr	Tr	Tr	Tr	Tr	0.2	0.2	0.2	0.2	0.3
α-Cadinol	1673	Tr	Tr	Tr	0.2	0.3	0.3	0.2	0.3	0.3	0.5
Total		99.1	99.0	99.4	99.3	98.5	98.3	98.7	98.1	97.8	97.9
Number of compounds quantified		21	21	21	25	27	27	27	28	26	28
Monoterpene hydrocarbons		13.5	13.4	14.1	11.8	14.1	14.1	12.8	14.0	13.3	14.6
Oxygenated monoterpenoids		0.9	1.0	0.9	0.9	1.2	1.4	1.4	1.7	1.5	1.6
Sesquiterpene hydrocarbons		15.1	15.5	12.3	13.8	18.3	21.3	17.3	20.5	19.9	20.2
Oxygenated sesquiterpenoids		0.3	0.3	0.2	0.8	1.1	1.0	0.7	1.4	1.0	1.3
Phenylpropanoids		68.8	68.1	70.9	71.2	63.2	59.9	66.0	59.9	61.8	59.4
Others		0.5	0.7	1.0	0.8	0.6	0.6	0.5	0.6	0.3	0.8

Note: ^a^ Order of compounds eluted on the HP-5MS column; ^b^ RI: retention index of compounds on the HP-5MS column; ^c^ Standard deviation was insignificant and excluded from the Table to avoid congestion (*n* = 3); Tr: Trace (concentration < 0.1%).

**Table 4:** Antimicrobial activity of essential oils of *Ocimum gratissimum* cultivated under different light conditions.

Formulas/Reference Compounds	Values (µg/mL)	The Concentration of Essential Oil Inhibiting the Tested Microorganisms (%)						
		Gram (+) Bacteria			Gram (−) Bacteria			Yeast
		*Staphylococcus aureus*	*Bacillus subtilis*	*Lactobacillus fermentum*	*Salmonella enterica*	*Escherichia coli*	*Pseudomonas aeruginosa*	*Candida albicans*
F1	IC_50_	>16,384	>16,384	>16,384	>16,384	6051 ± 16	>16,384	3220 ± 8.8 ^g^
	MIC	>16,384	>16,384	>16,384	>16,384	16,384	>16,384	8192
F2	IC_50_	>16,384	>16,384	>16,384	>16,384	>16,384	>16,384	2795 ± 15 ^d^
	MIC	>16,384	>16,384	>16,384	>16,384	>16,384	>16,384	8192
F3	IC_50_	>16,384	>16,384	>16,384	>16,384	>16,384	>16,384	3006 ± 10.6 ^g^
	MIC	>16,384	>16,384	>16,384	>16,384	>16,384	>16,384	8192
F4	IC_50_	>16,384	>16,384	>16,384	>16,384	>16,384	>16,384	3002 ± 12 ^g^
	MIC	>16,384	>16,384	>16,384	>16,384	>16,384	>16,384	8192
F5	IC_50_	>16,384	>16,384	>16,384	>16,384	>16,384	>16,384	2916 ± 8.7 ^e^
	MIC	>16,384	>16,384	>16,384	>16,384	>16,384	>16,384	8192
F6	IC_50_	>16,384	>16,384	>16,384	>16,384	>16,384	>16,384	3720 ± 10 ^h^
	MIC	>16,384	>16,384	>16,384	>16,384	>16,384	>16,384	16384
F7	IC_50_	>16,384	>16,384	>16,384	>16,384	>16,384	>16,384	2566 ± 9.8 ^c^
	MIC	>16,384	>16,384	>16,384	>16,384	>16,384	>16,384	4096
F8	IC_50_	>16,384	>16,384	>16,384	>16,384	>16,384	>16,384	2518 ± 9.0 ^b^
	MIC	>16,384	>16,384	>16,384	>16,384	>16,384	>16,384	4096
F9	IC_50_	>16,384	>16,384	>16,384	>16,384	>16,384	>16,384	2450 ± 14 ^a^
	MIC	>16,384	>16,384	>16,384	>16,384	>16,384	>16,384	4096
F10	IC_50_	>16,384	>16,384	>16,384	>16,384	>16,384	>16,384	2951 ± 21 ^f^
	MIC	>16,384	>16,384	>16,384	>16,384	>16,384	>16,384	8192
Ampicillin	IC_50_	0.02 ± 0.005	3.62 ± 0.15	1.03 ± 0.07				
	MIC	0.125 ± 0.0	32 ± 0.0	32 ± 0.0				
Cefotaxime	IC_50_				0.43 ± 0.05	0.007 ± 0.002	4.34 ± 0.15	
	MIC				32 ± 0.0	0.5 ± 0.0	8 ± 0.0	
Nystatin	IC_50_							1.32 ± 0.05
	MIC							8 ± 0.0

Note: Mean values followed by the same letter within a column are not statistically different for 0.05 significant level (*n* = 3). Statistical analyses were performed using IRRISTAT ver. 5.0 (International Rice Research Institute, Laguna, Philippines).

**Table 5:** Supplemental and control light conditions in cultivation of *Ocimum gratissimum*.

Formulas	Spectral Distribution	Duration (h/Day)	Lighting Time	Supplemental Light Intensity (µmol·m^−2^·s^−1^)	Total Daily Supplemental Light (mol·m^−2^·d^−1^)	Total Daily Light (mol·m^−2^·d^−1^)
F1	R:B:UVA ~ 71:20:9 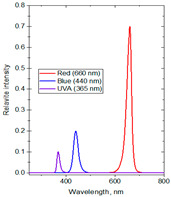	8	1:00–5:00 a.m. and 19:00–23:00 p.m.	100 ± 10	2.88	16.86
F2		6	2:00–5:00 a.m. and 19:00–22:00 p.m.	100 ± 10	2.16	16.14
F3		4	3:00–5:00 a.m. and 19:00–21:00 p.m.	100 ± 10	1.44	15.42
F4	R:B:G ~ 75:21:4 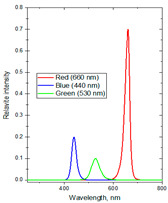	8	1:00–5:00 a.m. and 19:00–23:00 p.m.	100 ± 10	2.88	16.86
F5		6	2:00–5:00 a.m. and 19:00–22:00 p.m.	100 ± 10	2.16	16.14
F6		4	3:00–5:00 a.m. and 19:00–21:00 p.m.	100 ± 10	1.44	15.42
F7	R:B:G:FR ~ 43.5:43.5:8:5 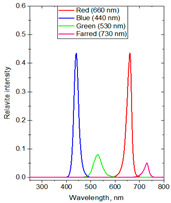	6	2:00–5:00 a.m. and 19:00–22:00 p.m.	120 ± 10	2.52	16.57
F8		6	2:00–5:00 a.m. and 19:00–22:00 p.m.	100 ± 10	2.16	16.14
F9		6	2:00–5:00 a.m. and 19:00–22:00 p.m.	80 ± 10	1.73	15.71
F10	Control	0	0	0	0	13.98

## Data Availability

All data are available in this publication.
